# First recorded hypertension and subsequent cardiovascular and kidney outcomes after newly diagnosed type 2 diabetes: a population-based cohort study

**DOI:** 10.3389/fendo.2026.1988998

**Published:** 2026-10-29

**Authors:** Yanyi Fu, Hanbin Cui, Hengyi Mao, Feng Sun, Lijia Liu, Fangfei Du, Jinming Yu, Sikun Chen

**Affiliations:** 1 School of Public Health, Fudan University, Shanghai, China; 2 Cardiology Center, The First Affiliated Hospital of Ningbo University, Ningbo, China; 3 Department of Epidemiology and Biostatistics, School of Public Health, Peking University, Beijing, China; 4 Key Laboratory of Major Disease Epidemiology, Ministry of Education (Peking University), Beijing, China; 5 Zhejiang Ningshu Health Big Data Operation Co., Ltd, Ningbo, China; 6 Wonders Information Co., Ltd, Shanghai, China

**Keywords:** cardiovascular diseases, electronic health records, hypertension, kidney diseases, type 2 diabetes

## Abstract

**Background:**

Coexistence of type 2 diabetes (T2D) and hypertension is associated with adverse cardiovascular and mortality outcomes. However, whether first recorded hypertension during the course of T2D marks a transition to a higher-risk phase remains uncertain. We examined subsequent outcomes and recorded assessments around hypertension recognition.

**Methods:**

Using regional electronic health records from Ningbo, China, we included 256,221 adults with a first recorded T2D diagnosis from 2012 through 2024 who had no recorded hypertension or major cardiorenal disease. We fitted Cox models with newly recorded hypertension as a time-varying exposure, adjusting for age, sex, calendar year, healthcare contact, comorbidities, medications, blood pressure, glycemia, lipids, kidney function, and liver enzymes. The primary outcome was the first occurrence of myocardial infarction, stroke, heart failure, chronic kidney disease, or all-cause death. Secondary outcomes included individual components above and a modified version of three-point major adverse cardiovascular events (3P-MACE), comprising myocardial infarction, stroke, and all-cause death. Among 107,946 patients with first recorded hypertension, assessment records were compared over six months before and after its recognition.

**Results:**

Among 256,221 adults, hypertension was first recorded during follow-up in 107,946 (42.13%). During a median follow-up of 4.11 years, 34,371 primary outcomes occurred. Incidence rates were 58.05 and 16.10 per 1,000 person-years during exposed and unexposed time, respectively (adjusted hazard ratio [HR] 2.49; 95% CI 2.43-2.55). The adjusted HR for modified 3P-MACE was 2.47 (95% CI 2.40–2.55). The primary association was strongest within 90 days of recognition (HR 3.37; 95% CI 3.10–3.65) and persisted beyond three years (HR 2.15; 95% CI 2.07–2.23). Outpatient-contact density was 17.42 versus 3.73 per person-year during exposed and unexposed time. Recorded urinary albumin-to-creatinine ratio (UACR) assessment increased from 3.35% to 4.15%, and assessment of both kidney function and UACR from 2.94% to 3.72%.

**Conclusions:**

First recorded hypertension identified a subsequent period of substantially higher observed cardiorenal and mortality risk, accompanied by limited increases in recorded assessment. New hypertension recognition may offer an opportunity to review existing cardiovascular and kidney assessments. Prospective studies should test whether structured reassessment at recognition improves care and outcomes.

## 1 Introduction

Type 2 diabetes (T2D) is a major and expanding cause of cardiovascular and kidney disease. The global number of people with diabetes is projected to reach 1.31 billion by 2050, with T2D accounting for 96.0% of the cases (1). Cardiovascular and kidney complications represent major long-term consequences of diabetes and are strongly associated with increased cardiovascular and all-cause mortality (2). In a multinational cohort of 772,336 people with T2D initially free of cardiovascular or renal disease, 18% developed a first cardiovascular or renal manifestation over a mean 4.5 years. Chronic kidney disease (CKD) and heart failure accounted for 36% and 24% of these first manifestations, respectively (3).

Cardiovascular and kidney risk in T2D is not static. The cardiovascular-kidney-metabolic (CKM) framework describes metabolic dysfunction, kidney injury, and cardiovascular disease as interconnected processes that evolve over time (4, 5). A risk assessment made when diabetes is first diagnosed may become outdated as new abnormalities emerge. Clinically recognizable events that mark such progression could provide opportunities to review whether recommended assessments have been completed.

Hypertension is a plausible marker event because it commonly develops during the course of T2D and shares many of its underlying pathways. In the Framingham Offspring Study, diabetes was associated with more than three-fold higher odds of incident hypertension over 7 years (6). Insulin resistance, renal sodium retention, arterial stiffness, endothelial dysfunction, inflammation, and excess adiposity may promote both rising blood pressure and cardiorenal injury (7). Thus, first recognized hypertension may represent both an additional hemodynamic burden and a visible manifestation of broader CKM progression, although the date of recording does not establish when elevated blood pressure began.

Previous studies have consistently shown that coexisting T2D and hypertension is associated with adverse cardiovascular and mortality outcomes (8, 9). Most studies, however, defined hypertension at baseline or focused on blood pressure levels or control, thereby characterizing a high-risk comorbid phenotype without locating when it emerged. One longitudinal study measured blood pressure at prespecified biennial examinations and found that progression to hypertensive blood pressure levels was associated with cardiovascular events among initially normotensive adults with diabetes (10). More recent studies have also linked an earlier age at hypertension diagnosis with greater cardiovascular and kidney risk in T2D (11, 12). Together, these findings indicate that both blood pressure progression and the timing of hypertension carry prognostic information. However, they do not establish whether the first recorded evidence of hypertension in routine care after newly diagnosed T2D can identify a subsequent higher-risk phase across cardiovascular, kidney, and mortality outcomes.

Currently, people with T2D are already recommended to undergo regular assessment of blood pressure, glycemia, lipids, kidney function, and albuminuria, including at least annual estimated glomerular filtration rate (eGFR) and urine albumin-creatinine ratio (UACR) assessment (2, 13, 14). However, evidence from China indicates that kidney disease screening remains incompletely implemented, with only 54.8% of patients having undergone CKD screening (15). Hypertension recognition may provide an additional clinically salient opportunity to review whether these assessments are up to date. But it remains uncertain whether the recognition is actually accompanied by more intensive cardiometabolic and kidney assessment in routine practice.

Using regional electronic health records (EHRs), we studied adults with newly recorded T2D who were initially free of recorded hypertension and major cardiorenal complications. We addressed two linked questions: (i) whether newly recognized hypertension, modeled as a time-varying exposure, could identify higher subsequent cardiorenal and mortality risk; and (ii) whether this transition is accompanied by changes in routine cardiometabolic and kidney assessment. Together, these analyses examine whether clinical care responds to a recognizable increase in risk and whether hypertension recognition may represent an opportunity for structured reassessment.

## 2 Methods

### 2.1 Study design and data source

This population-based cohort study used the Ningbo Regional Medical and Healthcare Database, which integrates longitudinal EHRs from 262 healthcare institutions in Ningbo, Zhejiang Province, China, including 23 tertiary hospitals, 23 secondary hospitals and 216 primary care or other healthcare institutions. The database contains person-level records of diagnoses, procedures, prescriptions, laboratory measurements, healthcare encounters, and deaths for more than 19 million individuals. Cohort entry began on 1 January, 2012, to provide a 365-day period in which baseline conditions, medication use, and measurements could be ascertained.

The study was conducted in accordance with the Declaration of Helsinki. The Medical Ethics Committee of the School of Public Health, Fudan University, approved the protocol (IRB00002408 and FWA00002399; approval H2026149) and waived individual informed consent because the study used routinely collected data without direct participant contact.

### 2.2 Study population

We identified adults aged at least 18 years whose first T2D diagnosis recorded anywhere in the Ningbo regional database (International Classification of Diseases, 10th Revision [ICD-10] code E11) occurred from 1 January 2012 through 31 December 2024. The date of that first record was the index date. The preceding 365 days constituted the baseline lookback period. Ascertainment of prior T2D diagnoses used all available historical database records and was not restricted to the cohort-entry period or the 365-day lookback period. To establish the clinical sequence of interest, eligible participants could have neither a hypertension diagnosis nor a prescription for any qualifying antihypertensive class before or on the index date. Diagnostic definitions are listed in **Supplementary Material**, **Supplementary Table S1**.

We excluded participants with evidence on or before the index date of myocardial infarction, stroke, heart failure, or CKD, including an eGFR below 60 mL/min/1.73 m^2^. We also excluded participants with no healthcare encounter after the index date, because subsequent exposure and outcome status could not be observed, and those missing more than three of the ten prespecified baseline measurements during the lookback period. Sequential exclusions are shown in **Figure 1**.

**Figure 1:**
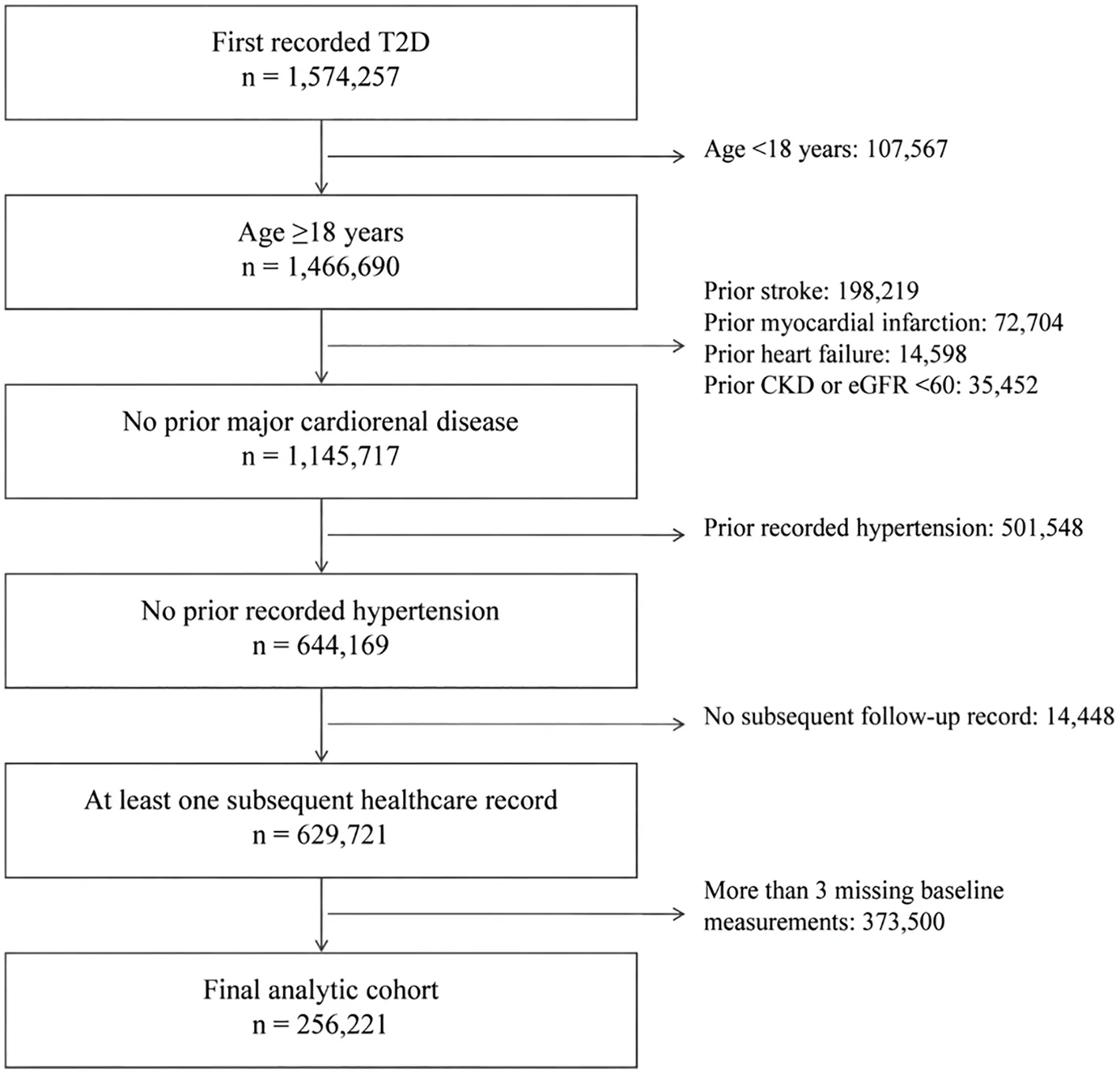
Flow diagram of participant selection. CKD, chronic kidney disease; eGFR, estimated glomerular filtration rate; T2D, type 2 diabetes.

### 2.3 Exposure

First recorded hypertension was defined as the first post-index record of either a hypertension diagnosis (ICD-10-CN code I10) or a prescription for antihypertensive medicines (Anatomical Therapeutic Chemical classes C02, C03, C07, C08, and C09, **Supplementary Table S1**). The earliest qualifying diagnosis or prescription defined the hypertension recognition date. Exposure was represented as a binary time-varying variable: each participant contributed unexposed person-time from the index date until the hypertension date and exposed person-time thereafter. Participants without a qualifying record remained unexposed throughout follow-up. Once hypertension was recorded, exposure status did not revert, irrespective of subsequent blood-pressure control or remission. This start-stop allocation preserved the temporal ordering of exposure and outcomes and avoided immortal time bias from classifying eventual hypertension status at baseline (16, 17).

### 2.4 Outcomes

The primary outcome was a prespecified cardiorenal composite outcome comprising acute myocardial infarction, stroke, heart failure, CKD, or all-cause death. Its composition was informed by published cardiorenal outcome frameworks and adapted to the outcomes ascertainable in the regional EHR database (18–20).

Secondary outcomes were each component analyzed separately. To examine outcomes less dependent on routine laboratory screening, we also analyzed a modified version of three-point major adverse cardiovascular events (3P-MACE), including myocardial infarction, stroke, or all-cause death, consistent with an approach used in a previous database study (21). Non-fatal outcomes were identified from diagnosis records. Death was obtained from the mortality records and death registry integrated in the database. The primary analysis disregarded outcome records occurring within 7 days after the T2D index date to reduce inclusion of previously unrecognized disease detected during the initial diagnostic work-up (22). Participants with an event during this blanking interval remained eligible for a later qualifying event. If hypertension and an outcome were recorded on the same date, the outcome was attributed to unexposed person-time and follow-up for that outcome ended on that date. No exposed person-time was accrued for that outcome.

For the primary cardiorenal composite outcome and modified 3P-MACE outcomes, follow-up ended at its first qualifying component, the last recorded healthcare encounter, or 30 June 2025. For each non-fatal component, follow-up ended at occurrence of the event, the last recorded healthcare encounter, death, or 30 June 2025. For all-cause mortality, follow-up ended at the last recorded healthcare encounter, death, or 30 June 2025.

### 2.5 Covariates

Baseline covariates were ascertained during the 365-day lookback period. When repeated measurements were available, the value closest to the index date was selected. The ten measurements were systolic blood pressure (SBP), diastolic blood pressure (DBP), fasting blood glucose (FBG), glycated hemoglobin (HbA1c), total cholesterol, high-density lipoprotein cholesterol (HDL-C), triglycerides, eGFR, alanine aminotransferase (ALT), and aspartate aminotransferase (AST). Additional covariates were age, sex, calendar year of the index date, number of baseline outpatient encounters, glucose-lowering medication, oral anticoagulant, non-steroidal anti-inflammatory drug, cerebrovascular disease other than stroke, peripheral vascular disease, and chronic obstructive pulmonary disease. Sex, medication use and comorbidities were coded as binary indicators. All continuous covariates, including index year, baseline measurements, age, and outpatient encounters, were modeled using restricted cubic splines (RCS) with four knots. In the missing-category sensitivity analysis, continuous covariates were categorized into quintiles, with a separate missing/unknown category for variables with missing values. All covariates were fixed at baseline. Coding definitions and adjustment rationale are provided in **Supplementary Table S1** and **Supplementary Methods**.

### 2.6 Recorded assessments around first recorded hypertension

Among the 107,946 participants with first recorded hypertension, we ascertained recorded outpatient contact and assessments during the 6 months before and after recognition. A complete 6-month pre-recognition observation window was required. The post-recognition window ended at 6 months or the end of the patients’ follow-up, whichever came first.

Assessment measures were selected based on the American Diabetes Association Standards of Care in Diabetes recommendations for routine evaluation of blood pressure, glycemic status, lipids, kidney function, and albuminuria in people with T2D (13, 14). These domains were represented by recorded SBP, HbA1c, any lipid assessment, serum creatinine/eGFR, and UACR, respectively, with outpatient visits additionally included as a measure of healthcare contact. Any lipid assessment comprised at least one measurement of total cholesterol, triglycerides, low-density lipoprotein cholesterol (LDL-C), or HDL-C. Duplicate lipid records on the same day counted once. We also ascertained complete kidney assessment, which was defined as at least one creatinine/eGFR assessment and at least one UACR assessment within the same window, without requiring them to occur on the same day.

### 2.7 Statistical analysis

Baseline characteristics were summarized as mean (standard deviation, SD), median (interquartile range, IQR), or number (percentage), as appropriate. We calculated event counts, person-time, median follow-up, and incidence rates with 95% confidence intervals (CIs) per 1,000 person-years. Person-time and events were allocated according to hypertension status. Crude incidence rate differences were calculated as the exposed rate minus the unexposed rate. For the primary composite, additional analyses restricted follow-up from the index date to 1,3, or 5 years to calculate the incidence rate differences.

Associations were estimated using Cox proportional hazards models in a start-stop data structure, with hypertension as a time-varying covariate. We report unadjusted and fully adjusted estimates, with all baseline covariates entered together in the model. Results are reported as hazard ratios (HRs) with 95% CIs. For non-fatal outcomes, including acute myocardial infarction, stroke, heart failure, and CKD outcomes, death was treated as a competing event and cause-specific HRs were estimated (23). The proportional hazards assumption was assessed using scaled Schoenfeld residuals. We additionally fitted piecewise Cox models with hypertension effects varying across five intervals since its first record: 0–90 days, 91–180 days, 181–365 days, >1–3 years, and >3 years.

For outpatient contact and assessments around hypertension recognition, we described the proportion with at least one record, the absolute change in percentage points with a 95% CI, and transitions between assessed and unassessed status. Paired binary completion was compared using McNemar’s test. We also calculated crude outpatient-contact and testing densities per person-year during time-varying unexposed and exposed person-time.

Prespecified subgroup analyses of the primary outcome were conducted by sex, age (<65 vs at least 65 years), baseline systolic blood pressure (<130 vs at least 130 mm Hg), total cholesterol (<5.2 vs at least 5.2 mmol/L), sex-specific high-density lipoprotein cholesterol (<1.0 mmol/L in men or <1.3 mmol/L in women vs higher), and baseline glucose-lowering medication use. Each subgroup model retained the full covariate set except the stratifying variable. Product terms between hypertension status and subgroup indicators were used to test heterogeneity.

We conducted the following sensitivity analyses to examine the robustness of the study findings: First, the post-index outcome blanking period was changed from 7 days to 0, 30, 90, or 180 days. Second, the hypertension exposure date was delayed by 7, 30, 90, or 180 days. Events arising during the lag were retained and allocated to unexposed person-time. This analysis assessed whether diagnostic clustering or an outcome occurring shortly after hypertension recognition explained the association (24). Third, because antihypertensive drugs may be used for other indications, the exposure was restricted to two independent hypertension diagnosis codes at different dates, with exposure beginning at the second record (25, 26). Fourth, all models were repeated among participants with complete data for every covariate. Fifth, we also fitted the missing-category model that treated the missing values as a separate missing/unknown category. Sixth, we also calculated E-values for the adjusted primary-composite HR and the confidence limit closest to the null to quantify the strength of unmeasured confounding needed to explain the association (27, 28).

Missing baseline covariate values were assumed to be missing at random (MAR), conditional on the observed information. Under this assumption, we used multiple imputation by chained equations with random-forest models to handle the missing covariate values. The imputation model included the variables used in the analytic models, and estimates from the imputed datasets were combined using Rubin’s rules (29). We also compared patients with complete and incomplete baseline measurements using standardized mean differences (SMDs).

Analyses were performed in R version 4.4.2 (R Foundation for Statistical Computing). All tests were two-sided, and P<0.05 was considered statistically significant. Reporting followed the STROBE and RECORD recommendations for observational studies using routinely collected health data (30).

## 3 Results

### 3.1 Participant characteristics

Of 1,574,257 individuals with a first recorded T2D diagnosis, 256,221 met the eligibility criteria (**Figure 1**). The mean age was 48.51 years (SD 14.36), and 148,513 (58.0%) were women, and 161,487 (63.0%) used glucose-lowering medications at baseline. Median baseline outpatient encounters were 6 (IQR 2-14). **Table 1** presents observed baseline characteristics for the full cohort. Complete data for all baseline covariates were available for 38,970 (15.2%) participants, and variable-specific missingness and characteristics by completeness are shown in **Supplementary Tables S2**, **S3**.

**Table 1:** Baseline characteristics.

Characteristic	Overall (N = 256,221)
Age, mean (SD), y	48.51 (14.36)
Male sex, No. (%)	107,708 (42.0)
Female sex, No. (%)	148,513 (58.0)
Outpatient encounters in look-back period, median (IQR)	6 (2–14)
Fasting blood glucose, mean (SD), mmol/L	7.96 (4.45)
Hemoglobin A1c, mean (SD), %	7.27 (3.10)
Systolic blood pressure, mean (SD), mm Hg	126.65 (16.41)
Diastolic blood pressure, mean (SD), mm Hg	77.97 (11.24)
Total cholesterol, mean (SD), mmol/L	5.16 (1.53)
Triglycerides, mean (SD), mmol/L	2.23 (2.44)
High-density lipoprotein cholesterol, mean (SD), mmol/L	1.38 (0.47)
Alanine aminotransferase, mean (SD), U/L	24.09 (59.75)
Aspartate aminotransferase, mean (SD), U/L	21.23 (58.08)
Estimated glomerular filtration rate, mean (SD), mL/min/1.73 m²	114.56 (48.36)
Oral anticoagulant use, No. (%)	1,128 (0.4)
Non-steroidal anti-inflammatory drug use, No. (%)	27,231 (10.6)
Glucose-lowering medication use, No. (%)	161,487 (63.0)
Cerebrovascular disease other than stroke, No. (%)	4,993 (1.9)
Peripheral vascular disease, No. (%)	318 (0.1)
Chronic obstructive pulmonary disease, No. (%)	13,830 (5.4)
Index calendar year, median (IQR)	2019 (2017–2022)

During follow-up, 107,946 patients (42.13%) had recorded hypertension. The median interval from the T2D index date to first recognition was 3.01 years. Across the hypertension-incidence risk set, 826,851 hypertension-free person-years accrued, corresponding to an incidence rate of first recorded hypertension of 130.55 per 1,000 person-years (95% CI 129.77-131.33).

### 3.2 Primary outcome

Over a median follow-up of 4.11 years (IQR 1.82-7.03), 34,371 primary cardiorenal composite outcomes occurred during 1,178,253 person-years, corresponding to an overall incidence rate of 29.17 per 1,000 person-years (95% CI 28.86-29.48). Of these events, 21,309 occurred during 367,095 hypertension-exposed person-years and 13,062 during 811,158 unexposed person-years. The respective rates were 58.05 (95% CI 57.27-58.83) and 16.10 (95% CI 15.83-16.38) per 1,000 person-years, yielding a crude rate difference of 41.95 (95% CI 41.12-42.77) per 1,000 person-years (**Table 2**). The rate differences were 81.60 (95% CI 77.70-85.50), 49.98 (95% CI 48.43-51.53), and 43.56 (95% CI 42.46-44.66) per 1,000 person-years at 1-, 3-, and 5-years of follow-up, respectively (**Supplementary Table S4**).

**Table 2:** Outcome events and incidence rates by time-varying recorded hypertension status.

Outcome	Unexposed events/PY	Unexposed IR (95% CI)	Exposed events/PY	Exposed IR (95% CI)	IRD (95% CI)
Primary composite outcome	13,062/ 811,158	16.10 (15.83–16.38)	21,309/ 367,095	58.05 (57.27–58.83)	41.95 (41.12–42.77)
Acute myocardial infarction	1,405/ 824,661	1.70 (1.62–1.80)	2,625/ 428,726	6.12 (5.89–6.36)	4.42 (4.17–4.67)
Stroke	4,918/ 819,164	6.00 (5.84–6.17)	8,451/ 405,573	20.84 (20.40–21.29)	14.83 (14.36–15.31)
Heart failure	2,179/ 824,591	2.64 (2.53–2.76)	6,221/ 421,397	14.76 (14.40–15.13)	12.12 (11.74–12.50)
Chronic kidney disease	2,866/ 822,924	3.48 (3.36–3.61)	6,445/ 426,972	15.09 (14.73–15.47)	11.61 (11.22–12.00)
All-cause death	2,827/ 826,432	3.42 (3.30–3.55)	6,850/ 443,539	15.44 (15.08–15.81)	12.02 (11.64–12.41)
Modified 3P-MACE	8,849/ 816,931	10.83 (10.61–11.06)	15,298/ 393,321	38.89 (38.28–39.52)	28.06 (27.41–28.72)

IRs and IRDs are per 1,000 person-years. IRDs are calculated as exposed minus unexposed rates. The primary composite outcome includes myocardial infarction, stroke, heart failure, CKD, and all-cause death. The modified 3P-MACE includes myocardial infarction, stroke, and all-cause death. IR, incidence rate; IRD, incidence rate difference; PY, person-year; CI, confidence interval; 3P-MACE, three-point major adverse cardiovascular events.

The 34,371 primary cardiorenal composite outcomes comprised 11,725 (34.1%) stroke events, 7,406 (21.5%) CKD events, 6,564 (19.1%) deaths, 6,304 (18.3%) heart failure events, and 3,218 (9.4%) myocardial infarction events. There were 788 patients (2.3%) with at least two components on the date of primary outcomes (**Supplementary Table S5**).

The unadjusted HR for the primary composite outcome was 4.20 (95% CI 4.10-4.30), and the fully adjusted HR was 2.49 (95% CI 2.43-2.55) (**Table 3**). In the fully adjusted model, hypertension-term and global proportional hazard tests had P<0.001 (**Supplementary Figure S1**). In the piecewise Cox model, HRs for the primary composite were 3.37 (95% CI 3.10-3.65) at 0–90 days, 3.15 (95% CI 2.86-3.48) at 91–180 days, 3.17 (95% CI 2.95-3.41) at 181–365 days, 2.51 (95% CI 2.40-2.61) at 1–3 years, and 2.15 (95% CI 2.07-2.23) beyond 3 years (**Table 4**).

**Table 3:** Associations of first recorded hypertension with the primary and secondary outcomes.

Outcome	Events	Unadjusted HR (95% CI)	Fully adjusted HR (95% CI)
Primary composite outcome	34,371	4.20 (4.10–4.30)	2.49 (2.43–2.55)
Acute myocardial infarction	4,030	4.76 (4.44–5.11)	2.73 (2.54–2.94)
Stroke	13,369	3.56 (3.42–3.70)	1.90 (1.83–1.98)
Heart failure	8,400	5.63 (5.34–5.93)	3.12 (2.95–3.29)
Chronic kidney disease	9,311	3.77 (3.59–3.95)	2.69 (2.55–2.83)
All-cause death	9,677	7.12 (6.79–7.47)	3.60 (3.43–3.79)
Modified 3P-MACE	24,147	4.53 (4.40–4.66)	2.47 (2.40–2.55)

Fully adjusted model was adjusted for age, sex, the number of outpatient encounters during the 365-day lookback period, calendar year of index date, baseline glucose-lowering medication, oral anticoagulant and non-steroidal anti-inflammatory drug use; cerebrovascular disease other than stroke, peripheral vascular disease, and chronic obstructive pulmonary disease; systolic and diastolic blood pressure, fasting glucose, glycated hemoglobin, total cholesterol, high-density lipoprotein cholesterol, triglycerides, estimated glomerular filtration rate, alanine aminotransferase, and aspartate aminotransferase. For non-fatal outcomes, HRs are cause-specific estimates with death treated as a competing event. CI, confidence interval; HR, hazard ratio; 3P-MACE, three-point major adverse cardiovascular event.

**Table 4:** Adjusted associations by time since first recorded hypertension.

Time	Primary composite HR (95% CI)	Myocardial infarction HR (95% CI)	Stroke HR (95% CI)	Heart Failure HR (95% CI)	CKD HR (95% CI)	All-cause death HR (95% CI)	Modified 3P-MACE HR (95% CI)
0–90 days	3.37 (3.10–3.65)	3.53 (2.71–4.59)	2.29 (1.91–2.73)	3.13 (2.51–3.91)	3.72 (2.98–4.66)	3.63 (3.22–4.10)	3.33 (3.03–3.65)
91–180 days	3.15 (2.86–3.48)	3.32 (2.54–4.33)	2.41 (1.99–2.93)	3.36 (2.65–4.25)	3.58 (2.77–4.62)	3.50 (2.99–4.11)	3.13 (2.80–3.50)
181–365 days	3.17 (2.95–3.41)	3.33 (2.74–4.04)	2.25 (1.97–2.57)	3.28 (2.75–3.92)	2.86 (2.39–3.43)	4.46 (3.92–5.07)	3.23 (2.97–3.51)
>1–3 years	2.51 (2.40–2.61)	2.69 (2.38–3.03)	1.97 (1.84–2.11)	3.03 (2.75–3.34)	2.55 (2.32–2.80)	3.70 (3.38–4.04)	2.52 (2.40–2.66)
>3 years	2.15 (2.07–2.23)	2.39 (2.13–2.67)	1.75 (1.66–1.85)	3.11 (2.88–3.36)	2.61 (2.45–2.79)	3.18 (2.92–3.47)	2.04 (1.95–2.14)

HRs were obtained by piecewise Cox models. The primary composite includes myocardial infarction, stroke, heart failure, CKD, and all-cause death. The modified 3P-MACE includes myocardial infarction, stroke, and all-cause death. Models use the full adjustment set including age, sex, the number of outpatient encounters during the 365-day lookback period, calendar year of index date, baseline glucose-lowering medication, oral anticoagulant and non-steroidal anti-inflammatory drug use; cerebrovascular disease other than stroke, peripheral vascular disease, and chronic obstructive pulmonary disease; systolic and diastolic blood pressure, fasting glucose, glycated hemoglobin, total cholesterol, high-density lipoprotein cholesterol, triglycerides, estimated glomerular filtration rate, alanine aminotransferase, and aspartate aminotransferase. CI, confidence interval; HR, hazard ratio; CKD, chronic kidney disease; 3P-MACE, three-point major adverse cardiovascular event.

### 3.3 Secondary outcomes

There were 4,030 acute myocardial infarctions, 13,369 strokes, 8,400 heart failure events, 9,311 CKD events, 9,677 deaths, and 24,147 modified 3P-MACE events during the corresponding outcome-specific follow-up periods (**Table 2**; **Supplementary Table S6**). In fully adjusted models, HRs were 2.73 (95% CI 2.54-2.94) for acute myocardial infarction, 1.90 (95% CI 1.83-1.98) for stroke, 3.12 (95% CI 2.95-3.29) for heart failure, 2.69 (95% CI 2.55–2.83) for CKD, 3.60 (95% CI 3.43–3.79) for all-cause death, and 2.47 (95% CI 2.40-2.55) for modified 3P-MACE (**Table 3**).

In the piecewise Cox model, the association between first recorded hypertension with myocardial infarction, stroke, CKD, and modified 3P-MACE attenuated with longer time since hypertension recognition but remained positive (**Table 4**).

### 3.4 Healthcare contact and recorded assessments

Among 107,946 patients with first recorded hypertension, the proportion with at least one outpatient visit increased from 84.36% in the preceding 6 months to 89.63% in the post-recognition window. Recorded lipid assessment increased from 46.85% to 50.42%, creatinine/eGFR assessment from 41.91% to 45.25%, SBP assessment from 24.22% to 27.09%, UACR assessment from 3.35% to 4.15%. HbA1c assessment decreased from 22.90% to 22.46%, an absolute change of -0.43 percentage points (95% CI -0.75 to -0.11). Complete kidney assessment increased from 3,176 (2.94%) to 4,019 (3.72%), corresponding to an increase of 0.78 percentage points (95% CI 0.65-0.91) (**Table 5**; **Supplementary Tables S7**, **S8**).

**Table 5:** Recorded assessments within 6 months before and after first recorded hypertension.

Assessment	Before, n (%)	After, n (%)	Change in percentage points (95% CI)
Outpatient visit	91,059 (84.36)	96,756 (89.63)	5.28 (5.03–5.52)
Any lipid assessment	50,569 (46.85)	54,428 (50.42)	3.57 (3.19–3.95)
Creatinine/eGFR	45,240 (41.91)	48,845 (45.25)	3.34 (2.97–3.70)
SBP	26,143 (24.22)	29,241 (27.09)	2.87 (2.63–3.11)
HbA1c	24,715 (22.90)	24,249 (22.46)	−0.43 (−0.75 to −0.11)
UACR	3,611 (3.35)	4,475 (4.15)	0.80 (0.66–0.94)
Complete kidney assessment	3,176 (2.94)	4,019 (3.72)	0.78 (0.65–0.91)

Completion means at least one record in the specified window. Any lipid assessment includes at least one measurement of total cholesterol, triglycerides, low-density lipoprotein cholesterol, or high-density lipoprotein cholesterol. Complete kidney assessment requires both creatinine/eGFR and UACR within the same window.

Across time-varying exposure states, outpatient-contact density was 17.42 per person-year during exposed time and 3.73 during unexposed time among the whole cohort of 256,221 patients. Assessment densities were higher during exposed than unexposed person-time, with rate differences of 0.441 per person-year for HbA1c tests, 1.275 per person-year for lipid assessment, 1.183 per person-year for creatinine/eGFR assessment, 0.070 per person-year for UACR assessment, and 3.244 per person-year for SBP assessment (**Supplementary Table S9**).

### 3.5 Sensitivity and subgroup analyses

Changing the outcome blanking period did not materially alter the primary estimate: the fully adjusted HR was 2.49 (95% CI 2.43–2.55) with no blanking, 2.47 (95% CI 2.41–2.53) with a 30-day blanking period, 2.43 (95% CI 2.36-2.49) with a 90-day blanking period, and 2.39 (95% CI 2.33-2.45) with a 180-day blanking period (**Supplementary Table S10**). Delaying hypertension exposure by 7, 30, 90, 180 days attenuated the estimate to 2.31 (95% CI 2.26-2.37), 2.12 (95% CI 2.07-2.18), 1.90 (95% CI 1.85-1.95), and 1.75 (95% CI 1.71-1.80), respectively (**Supplementary Table S11**). Restricting exposure to two diagnosis codes yielded an HR of 1.73 (95% CI 1.69–1.78; **Supplementary Table S12**).

Among 38,970 complete cases, 5,219 primary events were observed, and the adjusted HR was 2.49 (95% CI 2.34-2.66), and component-specific estimates remained directionally consistent (**Supplementary Table S13**). The missing-category analysis yielded an HR of 2.52 (95% CI 2.46-2.59; **Supplementary Table S14**). The primary-composite E-value was 4.42 for the point estimate and 4.29 for its lower confidence limit.

The association between first recorded hypertension and the primary outcome remained positive across all prespecified subgroups (**Figure 2**; **Supplementary Table S15**). The primary outcome HR was 2.80 (95% CI 2.71-2.89) at age <65 years and 2.10 (95% CI 2.02-2.19) at age ≥65 years, and 2.68 (95% CI 2.58–2.78) with baseline SBP <130 mm Hg and 2.37 (95% CI 2.29–2.45) with higher pressure. HRs were 2.47 (95% CI 2.39–2.55) in men and 2.58 (95% CI 2.48–2.68) in women. All full-cohort interaction tests had P<0.001.

**Figure 2:**
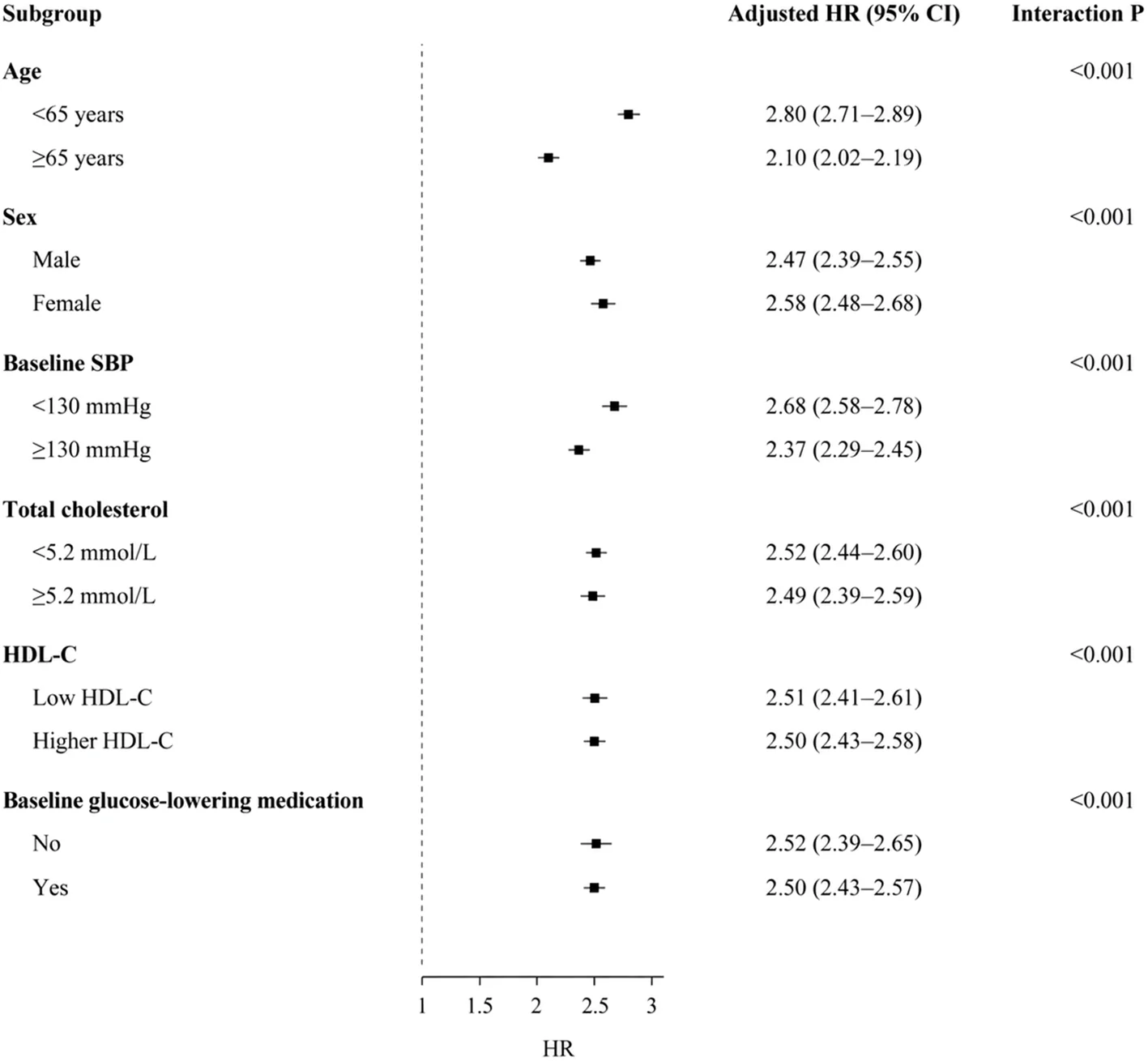
Subgroup associations with the primary composite outcome. Points represent hazard ratios and horizontal lines represent 95% confidence intervals. HDL-C, high-density lipoprotein cholesterol; HR, hazard ratio; SBP, systolic blood pressure; T2D, type 2 diabetes.

## 4 Discussion

In this population-based cohort of 256,221 adults with newly diagnosed T2D, first recorded hypertension identified a distinctly higher-risk phase in the subsequent course of T2D. Forty-two percent of patients had first recorded hypertension during follow-up. After its recognition, the primary cardiorenal composite outcome occurred at 58.05 per 1,000 person-years, compared with 16.10 during unexposed person-time, and the fully adjusted hazard was more than twofold higher (HR 2.49). Associations remained significant across acute myocardial infarction, stroke, heart failure, CKD, all-cause death, and modified 3P-MACE. The excess risk was particularly pronounced early after recognition but remained evident beyond 3 years. The proportion of participants receiving recorded cardiometabolic and kidney assessments increased after hypertension recognition, although the increases were generally modest, particularly for UACR. Hypertension recognition therefore appears to identify an important transition to a higher-risk state accompanied by limited intensification of routine risk assessment.

The overall association is consistent with studies of established diabetes-hypertension comorbidity. Concurrent hypertension and T2D have been associated with higher cardiovascular and mortality risks (8, 9), while observational studies have shown graded associations of baseline systolic blood pressure with macrovascular and microvascular complications in T2D (31, 32). A longitudinal study also found that progression from normotension to hypertension between two scheduled examinations was associated with cardiovascular events (10). Building on this work, we examined the timing of first hypertension recognition in routine care as a clinically observable transition point, and extended the assessment to a broader range of outcomes, including acute myocardial infarction, stroke, heart failure, chronic kidney disease, and all-cause mortality.

The findings also accord with evidence that longitudinal blood pressure burden contains information not captured by a single baseline measurement. In the Rio de Janeiro T2D cohort, higher cumulative ambulatory blood pressure during treatment was associated with adverse macrovascular and microvascular outcomes (33). Greater visit-to-visit blood pressure variability has likewise been associated with incident heart failure, and with subsequent hypertension and deterioration in renal function among people with T2D (34, 35). Although these studies did not examine incident hypertension, they support the broader interpretation that blood pressure–related risk evolves during the course of T2D and is not fully characterized by baseline blood pressure alone. In this context, the first clinical recognition of hypertension may represent a clinically visible transition within an evolving blood pressure trajectory.

Several interconnected mechanisms may explain why newly recognized hypertension marks a higher-risk state. The CKM framework describes metabolic dysfunction, vascular injury, kidney dysfunction, and cardiovascular disease as mutually reinforcing processes (4, 5). In T2D, insulin resistance, excess adiposity, endothelial dysfunction, oxidative stress, inflammation, sodium retention, and renin-angiotensin-aldosterone system activation may promote both rising blood pressure and organ injury (7, 36, 37). Once hypertension develops, higher afterload can contribute to left ventricular hypertrophy, fibrosis, and diastolic dysfunction, while higher systemic and intraglomerular pressure can aggravate albumin leakage, podocyte injury, and nephron loss (7, 36, 38). Early cardiac or renal abnormalities may in turn facilitate hypertension through arterial stiffness, neurohormonal activation, and volume expansion. Thus, newly recorded hypertension may be both a contributor to subsequent injury and a marker that broader CKM abnormalities have progressed. This dual role may explain the broad association observed between hypertension and adverse cardiorenal outcomes.

Associations were also observed in every prespecified subgroup, and the relative association was stronger in adults younger than 65 years, women, and participants not using glucose-lowering medication at baseline. The age and sex findings are consistent with reports that an earlier age at hypertension diagnosis carries greater residual cardiovascular and mortality risk in T2D, particularly among women (8, 11, 39). Statistically significant interactions were also observed across subgroups defined by total cholesterol, HDL-C, and baseline glucose-lowering medication use, but the between-stratum differences in HRs were modest and unlikely to be clinically meaningful.

Current guidelines recommend periodic assessment of blood pressure, glycemic status, lipids, eGFR, and UACR in patients with T2D (13, 14). Our findings suggest that newly recorded hypertension identifies a higher cardiorenal risk state and that its recognition may provide an opportunity to review evolving risk and recent assessment results. However, only modest changes in assessment occurred after hypertension recognition in our study, suggesting a risk-care mismatch. Studies of other newly diagnosed hypertension populations also indicate incomplete assessment coverage during the initial months after diagnosis. In Alberta, Canada, around 69.5%, 54.3%, and 53.7% of patients underwent eGFR, LDL-C, and fasting glucose or HbA1c testing, respectively, within the first 12 months after diagnosis (40). In a selected US cohort that excluded patients with any target laboratory testing during the preceding six months, only 42.7% received a metabolic panel and 17.0% underwent lipid testing within six months after diagnosis (41). The current findings together suggest that hypertension recognition does not consistently coincide with broad laboratory assessment coverage across care settings.

Substantial gaps in recorded assessment coverage persisted when records from both sides of recognition were considered. Across a span of up to 12 months, comprising 6 months before and after the first recorded hypertension, our results showed that 30-40% of patients had no recorded lipid or renal function assessment in either window, 60-70% lacked SBP or HbA1c assessment records, and particularly more than 90% had no UACR records. Thus, appreciable numbers remained without recorded assessment in individual cardiorenal risks around a transition marking higher risk.

Cardiorenal assessments varied substantially across care settings. Across 24 US healthcare organizations, median annual eGFR and HbA1c testing rates could reach 89.5% and 91.1%, respectively (42), and annual HbA1c monitoring in northern Thai primary care ranged from 79.7% to 88.6% (43). Conversely, in Chongqing, China, a community survey reported annual coverage of only 8% for HbA1c, 54% for lipids, and 45% for nephropathy screening (44). Among the selected assessments, UACR was the most pronounced remaining gap, with coverage ranging merely from 3.35% to 4.15%. Accordingly, albuminuria assessment deserves particular attention. Even in the same US organizations with high blood-test coverage, median annual UACR testing was only 52.9% (42). Similarly, a Dutch primary care cohort reported annual UACR testing rates of 45.2-57.4% among patients with T2D (45). Chinese studies also suggest incomplete UACR assessment in some care settings. In a Shanghai community diabetes study, coverage of UACR screening was only around 26-27% (46). In a broader Chinese multicenter cohort of patients with CKD, UACR results were available for only 7.0% at baseline, further illustrating the limited documentation of albuminuria assessment even among patients with established kidney dysfunction (47). However, the distinction between kidney function and albuminuria assessment is clinically important because albuminuria may become apparent before eGFR declines and provides additional information about cardiorenal risks (48), and guidelines therefore recommend both measures (14). The very low recorded UACR coverage suggests a substantial implementation gap and highlights hypertension recognition as an opportunity to review whether both kidney function and albuminuria assessments are up to date.

This study has several strengths. Data covered a large cohort from a large regional EHR database in the routine care settings. The time-varying exposure definition could help more precisely estimate the cardiorenal risk transitions since the first recorded hypertension and avoid immortal time bias.

However, several limitations should also be considered. First, EHRs capture clinical recognition rather than biological onset. Hypertension and T2D may have been present but undiagnosed, and qualifying antihypertensive medications may occasionally have been prescribed for other indications. The stricter diagnosis-based sensitivity analysis reduced but did not eliminate exposure misclassification. Exposure status also remained positive after recognition, irrespective of subsequent blood-pressure control or remission, and changes in treatment and adherence were not modeled. Second, increased healthcare contact and testing after hypertension recognition may have increased opportunities to detect previously unrecognized cardiorenal disease. Conversely, emerging cardiorenal abnormalities may have prompted hypertension recognition. Adjustment for the number of outpatient encounters during the baseline lookback period reduced, but could not eliminate, this concern. Exposure-lag analyses found attenuation with longer lags, suggesting that diagnostic clustering and reverse causation may contribute to the observed associations, although the associations persisted for outcomes less dependent on routine screening including myocardial infarction, stroke, and death.

Third, baseline measurements had substantial missingness, particularly SBP, DBP, and HbA1c. Most observed baseline characteristics were broadly comparable between participants with complete and incomplete measurements, although modest differences were observed in age, index year, and baseline outpatient contact, suggesting that measurement availability was related to patient characteristics and care patterns. Multiple imputation relied on the MAR assumption conditional on the observed information included in the imputation model. Similar primary-composite estimates across imputed, complete-case, and missing-category analyses provide some reassurance about the robustness of the findings but cannot fully establish the MAR assumption. Fourth, residual confounding is likely because adiposity, albuminuria, smoking, alcohol use, dietary sodium, physical activity, family history, and socioeconomic circumstances were unavailable or incomplete. For example, excess adiposity may contribute to both rising blood pressure and cardiorenal injury, and incomplete adjustment for such shared risk factors may partly explain the observed associations. E-values were used to quantify sensitivity to unmeasured confounding.

Fifth, outcomes were identified from routine records in a single region in eastern China. Events treated outside the regional system may have been missed, and generalizability to other healthcare systems and populations may be limited. Besides, diagnosis may be miscoded without a laboratory-confirmed definition, which may contribute to outcome misclassification. Sixth, recorded tests are proxies for assessment processes and cannot establish whether comprehensive risk assessment was completed or clinically appropriate. Incomplete recording may underestimate assessment coverage. Finally, the observational design precludes causal inference. The current study examined the association between first recorded hypertension and subsequent risk, alongside changes in recorded assessment patterns around recognition. The study did not directly compare a defined reassessment strategy with usual care. Hence, future prospective studies are needed to directly compare a structured reassessment and management pathway initiated at a first confirmed hypertension diagnosis with usual care, and evaluate its effects on long-term cardiorenal outcomes.

In conclusion, in this population-based regional EHR cohort, newly recorded hypertension after the first recorded T2D diagnosis identified a subsequent period with markedly higher observed rates and hazards of major cardiovascular and kidney outcomes and death. Although routine cardiometabolic and kidney assessments generally increased around this transition, the increases in assessment coverage were modest and important gaps persisted. Newly recorded hypertension may therefore represent an underused opportunity for structured cardiorenal risk reassessment. However, prospective implementation and interventional studies are required to determine whether acting at this time point improves care and clinical outcomes.

## Data Availability

The data analyzed in this study is subject to the following licenses/restrictions: The data that support the findings of this study contain sensitive clinical information and are not publicly available because of ethical and data-protection restrictions. Subject to a formal data-use agreement and approval by the relevant data access committee, qualified researchers may apply for controlled access to de-identified data for the purpose of reproducing the study findings or conducting approved secondary analyses. Requests to access these datasets should be directed to Sikun Chen, chensikun1232010@gmail.com.
