# Supplementary material for: First recorded hypertension and subsequent cardiovascular and kidney outcomes after newly diagnosed type 2 diabetes: a population-based cohort study
Source: Front Endocrinol (Lausanne). 2026 Oct 29;17:1988998. doi: 10.3389/fendo.2026.1988998 (PMC13754687; doi:10.3389/fendo.2026.1988998)
Supplement: Supplementary file 1 [file SupplementaryFile1.docx]

**Supplementary Material**

**Adjustment rationale**

Age, sex, and index year represent demographic and calendar-time variation in recorded hypertension and outcomes. Baseline outpatient encounters capture opportunities for diagnosis and differences in health status. Baseline medications and comorbidities characterize clinical burden. Blood pressure, glucose, glycated hemoglobin (HbA1c), lipids, kidney function, and liver enzymes provide additional baseline risk information. Blood pressure and estimated glomerular filtration rate (eGFR) may already reflect the disease process preceding clinical recognition. The fully adjusted model therefore estimates an association conditional on measured baseline characteristics, rather than a causal effect of hypertension onset or treatment. Post-recognition tests and visits were described as care processes and were not added to the main adjustment set, because they may respond to recognition, symptoms, or evolving disease.

All continuous covariates, including age, index year, and baseline outpatient encounters, were modeled using restricted cubic splines (RCS) with four knots placed at the 5^th^, 35^th^, 65^th^, 95^th^ percentiles to allow nonlinear associations with outcomes.

**Multiple imputation and sensitivity analyses for missing data**

The primary analysis used multiple imputation by chained equations with random-forest models and combined model estimates using Rubin’s rules. The working assumption was missing at random conditional on observed information included in the imputation model. Twenty imputed datasets were generated, with 20 iterations per dataset.

To assess the robustness of the estimates obtained after imputation, outcome models were repeated in complete cases and with a missing-category approach. In the latter analysis, continuous covariates were categorized into quintiles, with a separate missing/unknown category for variables with missing values. Similar estimates across these analyses do not verify the missing-at-random assumption or remove selection bias in complete cases.

**Recorded assessment processes**

For the paired analyses, N = 107,946 participants with first recorded hypertension had a complete preceding 6-month observation window. The subsequent window ended at 6 months after newly recorded hypertension or the end of follow-up. A missing assessment record was counted as no recorded assessment. Any lipid assessment comprised total cholesterol, triglycerides, low-density lipoprotein cholesterol (LDL-C), or high-density lipoprotein cholesterol (HDL-C), with same-day duplicate lipid records counted once. Kidney-function assessment included serum creatinine or eGFR. Complete kidney assessment required kidney-function assessment and urinary albumin-to-creatinine ratio (UACR) in the same window. Absolute changes and paired transition counts describe recording in these windows and do not establish whether clinical review was appropriate or complete.

**E-value interpretation**

E-values quantify the minimum strength of association, on the risk-ratio scale, that unmeasured confounding would need to have with both recorded hypertension and the outcome to explain the estimated association after measured adjustment. We report a direct approximation treating the HR as a risk ratio. The calculations use the overall adjusted HR and its lower confidence limit. The relevant methodological references are listed in the main manuscript.

**Table S1. Diagnostic and medication codes used in the study**

| **Condition or medication** | **ICD-10-CN or ATC codes** |
| --- | --- |
| T2D | E11 |
| Hypertension | I10 |
| CKD | N18 |
| Pre-existing myocardial infarction (baseline exclusion) | I21; I22; I23; I25.2 |
| Myocardial infarction | I21; I22 |
| Stroke | I60; I61; I62; I63; I64 |
| Heart failure | I50 |
| Cerebrovascular disease other than stroke | G45; I65; I66; I67; I68 |
| Peripheral vascular disease | I70; I72.1; I72.3; I72.4; I72.9; I73; I74.2; I74.3; I74.4; I74.5 |
| Chronic obstructive pulmonary disease | J41; J42; J43; J44 |
| Antihypertensive medication | C02; C03; C07; C08; C09 |
| Oral anticoagulant | B01AA; B01AE; B01AF |
| Non-steroidal anti-inflammatory medication | M01A |
| Glucose-lowering medication | A10 |

Diagnoses use International Classification of Diseases, 10th Revision, Chinese modification (ICD-10-CN) codes; medications use Anatomical Therapeutic Chemical (ATC) codes. Antihypertensive classes are C02 (other antihypertensives), C03 (diuretics), C07 (beta blockers), C08 (calcium-channel blockers), and C09 (agents acting on the renin–angiotensin system). The main exposure begins at the first qualifying diagnosis or prescription after type 2 diabetes (T2D) diagnosis.

**Table S2. Measurement-specific missingness**

| **Measurement** | **Missing, n** | **Missing, %** | **Observed, n** |
| --- | --- | --- | --- |
| HbA1c | 138,719 | 54.14 | 117,502 |
| Systolic blood pressure (SBP) | 124,853 | 48.73 | 131,368 |
| Diastolic blood pressure (DBP) | 124,754 | 48.69 | 131,467 |
| eGFR | 37,238 | 14.53 | 218,983 |
| Triglycerides | 12,073 | 4.71 | 244,148 |
| Aspartate aminotransferase (AST) | 10,292 | 4.02 | 245,929 |
| HDL-C | 5,442 | 2.12 | 250,779 |
| Total cholesterol | 3,902 | 1.52 | 252,319 |
| Alanine aminotransferase (ALT) | 2,546 | 0.99 | 253,675 |
| Fasting blood glucose (FBG) | 946 | 0.37 | 255,275 |

Analytic cohort N = 256,221. Complete measurements were available for 38,970 participants (15.2%), and 217,251 (84.8%) had at least one missing measurement. All other listed baseline covariates were recorded for the full cohort.

**Table S3. Complete versus incomplete baseline measurements**

| **Characteristic** | **Complete (n = 38,970)** | **Incomplete (n = 217,251)** | **SMD** |
| --- | --- | --- | --- |
| Age, mean (SD), y | 51.48 (14.37) | 47.97 (14.29) | 0.245 |
| SBP, mean (SD), mm Hg | 125.77 (16.35) | 127.01 (16.42) | 0.076 |
| DBP, mean (SD), mm Hg | 77.47 (11.10) | 78.19 (11.30) | 0.064 |
| FBG, mean (SD), mmol/L | 8.26 (4.21) | 7.90 (4.49) | 0.081 |
| HbA1c, mean (SD), % | 7.22 (3.07) | 7.30 (3.12) | 0.025 |
| Total cholesterol, mean (SD), mmol/L | 5.26 (1.33) | 5.15 (1.56) | 0.076 |
| HDL-C, mean (SD), mmol/L | 1.38 (0.44) | 1.38 (0.48) | 0.001 |
| Triglycerides, mean (SD), mmol/L | 2.14 (2.03) | 2.24 (2.51) | 0.047 |
| ALT, mean (SD), U/L | 23.77 (41.10) | 24.14 (62.54) | 0.007 |
| AST, mean (SD), U/L | 20.92 (39.53) | 21.29 (60.95) | 0.007 |
| eGFR, mean (SD), mL/min/1.73 m² | 112.42 (52.98) | 115.02 (47.26) | 0.052 |
| Calendar year of index date, mean (SD) | 2019.87 (2.98) | 2019.01 (3.24) | 0.277 |
| Outpatient encounters in look-back period, mean (SD) | 10.99 (11.60) | 9.44 (10.73) | 0.139 |
| Female sex, n (%) | 21,994 (56.4) | 126,519 (58.2) | 0.036 |
| Glucose-lowering medication use, n (%) | 24,717 (63.4) | 136,770 (63.0) | 0.010 |
| Oral anticoagulant use, n (%) | 249 (0.6) | 879 (0.4) | 0.033 |
| Non-steroidal anti-inflammatory drug use, n (%) | 4,102 (10.5) | 23,129 (10.6) | 0.004 |
| Cerebrovascular disease other than stroke, n (%) | 1,028 (2.6) | 3,965 (1.8) | 0.055 |
| Peripheral vascular disease, n (%) | 70 (0.2) | 248 (0.1) | 0.017 |
| Chronic obstructive pulmonary disease, n (%) | 2,340 (6.0) | 11,490 (5.3) | 0.031 |

Continuous measurements are mean (SD). Categorical measurements are n (%). Incomplete-group summaries use observed values for each measurement. Missingness was related to observed age, calendar year, and outpatient contact.

**Table S4. Primary-composite rates within 1-, 3-, and 5-years of follow-up**

|  | **State** | **Events** | **PY** | **IR (95% CI)** |
| --- | --- | --- | --- | --- |
| ≤1 year | Unexposed | 4,692 | 205,684.14 | 22.81 (22.16–23.46) |
| ≤1 year | Exposed | 2,829 | 27,094.12 | 104.41 (100.57–108.26) |
| ≤3 years | Unexposed | 8,671 | 490,801.00 | 17.67 (17.30–18.04) |
| ≤3 years | Exposed | 7,778 | 114,978.00 | 67.65 (66.14–69.15) |
| ≤5 years | Unexposed | 10,921 | 660,584.80 | 16.53 (16.22–16.84) |
| ≤5 years | Exposed | 12,477 | 207,623.40 | 60.09 (59.04–61.15) |

Exposed and unexposed person-time were allocated by time-varying recorded-hypertension status. IRs are per 1,000 PY. CIs use Wald Poisson approximations.

**Table S5. Components recorded on the first primary-composite event date**

| **Component or overlap** | **Participants** | **Percentage** |
| --- | --- | --- |
| Stroke | 11,725 | 34.11 |
| CKD | 7,406 | 21.55 |
| All-cause death | 6,564 | 19.10 |
| Heart failure | 6,304 | 18.34 |
| Myocardial infarction | 3,218 | 9.36 |
| Same-day multiple-component patients | 788 | 2.29 |

Component counts are not mutually exclusive. Their sum is 35,217 because 788 participants had at least two components recorded on the first composite-event date. The 846 excess component records are not the count of people with overlap, because some people had more than two components.

**Table S6. Outcome-specific follow-up and total events**

| **Outcome** | **Follow-up, years Median (IQR)** | **PY** | **Events** |
| --- | --- | --- | --- |
| Primary composite | 4.11 (1.82–7.03) | 1,178,253 | 34,371 |
| Myocardial infarction | 4.47 (1.99–7.44) | 1,253,386 | 4,030 |
| Stroke | 4.33 (1.93–7.28) | 1,224,738 | 13,369 |
| Heart failure | 4.44 (1.99–7.39) | 1,245,988 | 8,400 |
| CKD | 4.46 (1.99–7.42) | 1,249,896 | 9,311 |
| All-cause death | 4.55 (2.03–7.52) | 1,269,971 | 9,677 |
| Modified 3P-MACE | 4.26 (1.89–7.20) | 1,210,253 | 24,147 |

Follow-up is calculated from the T2D index date to the outcome-specific event or censoring. Component analyses are followed to their own first events.

**Table S7. Paired completion before and after first recorded hypertension**

| **Assessment** | **Neither window** | **After only** | **Before only** | **Both windows** |
| --- | --- | --- | --- | --- |
| Outpatient visit | 4,912 | 11,975 | 6,278 | 84,781 |
| Any lipid assessment | 32,476 | 24,901 | 21,042 | 29,527 |
| Creatinine/eGFR | 40,640 | 22,066 | 18,461 | 26,779 |
| SBP | 71,111 | 10,692 | 7,594 | 18,549 |
| HbA1c | 68,198 | 15,033 | 15,499 | 9,216 |
| UACR | 100,889 | 3,446 | 2,582 | 1,029 |
| Complete kidney assessment | 101,634 | 3,136 | 2,293 | 883 |

Complete kidney assessment requires both creatinine/eGFR and UACR within the same window.

**Table S8. Number of recorded visits or assessments in each window**

| **Assessment** | **Before: 0 / 1 / ≥2 (%)** | **After: 0 / 1 / ≥2 (%)** |
| --- | --- | --- |
| Outpatient visit | 15.6 / 13.2 / 71.2 | 10.4 / 6.33 / 83.3 |
| Any lipid assessment | 53.2 / 29.5 / 17.3 | 49.6 / 26.1 / 24.3 |
| Creatinine/eGFR | 58.1 / 24.9 / 17.0 | 54.8 / 22.2 / 23.0 |
| SBP | 75.8 / 8.77 / 15.4 | 72.9 / 7.31 / 19.8 |
| HbA1c | 77.1 / 18.0 / 4.90 | 77.5 / 16.7 / 5.74 |
| UACR | 96.7 / 2.94 / 0.401 | 95.9 / 3.32 / 0.824 |

**Table S9. Healthcare-contact and testing density by time-varying exposure state**

| **Contact or test** | **Unexposed records** | **Unexposed rate / PY** | **Exposed records** | **Exposed rate / PY** | **Rate difference** |
| --- | --- | --- | --- | --- | --- |
| Outpatient visits | 3,026,214 | 3.731 | 6,395,279 | 17.421 | 13.690 |
| HbA1c tests | 127,885 | 0.158 | 219,743 | 0.599 | 0.441 |
| Any lipid assessments | 362,886 | 0.447 | 631,993 | 1.722 | 1.275 |
| Creatinine/eGFR assessments | 334,200 | 0.412 | 585,574 | 1.595 | 1.183 |
| UACR assessments | 14,163 | 0.018 | 32,171 | 0.088 | 0.070 |
| SBP assessments | 391,408 | 0.483 | 1,368,067 | 3.727 | 3.244 |

Denominators for the care-process density analysis are 811,158 unexposed PY and 367,095 exposed PY, corresponding to the primary-outcome person-time. Rates count encounters or assessments per PY.

**Table S10. Sensitivity to the outcome blanking interval after T2D diagnosis**

| **Outcome** | **0 days** | **30 days** | **90 days** | **180 days** |
| --- | --- | --- | --- | --- |
| Primary composite | 2.49 (2.43–2.55) | 2.47 (2.41–2.53) | 2.43 (2.36–2.49) | 2.39 (2.33–2.45) |
| Myocardial infarction | 2.72 (2.52–2.92) | 2.73 (2.54–2.94) | 2.72 (2.52–2.93) | 2.70 (2.49–2.92) |
| Stroke | 1.91 (1.83–1.98) | 1.89 (1.82–1.97) | 1.88 (1.81–1.96) | 1.86 (1.78–1.94) |
| Heart failure | 3.12 (2.95–3.30) | 3.11 (2.94–3.29) | 3.09 (2.92–3.27) | 3.07 (2.90–3.26) |
| CKD | 2.68 (2.55–2.82) | 2.66 (2.53–2.80) | 2.64 (2.50–2.78) | 2.60 (2.47–2.75) |
| All-cause death | 3.60 (3.43–3.78) | 3.61 (3.43–3.80) | 3.54 (3.35–3.74) | 3.52 (3.32–3.73) |
| Modified 3P-MACE | 2.48 (2.41–2.56) | 2.45 (2.38–2.53) | 2.39 (2.32–2.47) | 2.34 (2.27–2.42) |

**Table S11. Sensitivity to delayed exposure classification after first recorded hypertension**

| **Outcome** | **7-day lag** | **30-day lag** | **90-day lag** | **180-day lag** |
| --- | --- | --- | --- | --- |
| Primary composite | 2.31 (2.26–2.37) | 2.12 (2.07–2.18) | 1.90 (1.85–1.95) | 1.75 (1.71–1.80) |
| Myocardial infarction | 2.59 (2.40–2.78) | 2.43 (2.25–2.61) | 2.12 (1.97–2.29) | 1.88 (1.75–2.03) |
| Stroke | 1.77 (1.70–1.84) | 1.68 (1.62–1.75) | 1.60 (1.54–1.67) | 1.52 (1.46–1.59) |
| Heart failure | 2.89 (2.73–3.05) | 2.68 (2.54–2.84) | 2.38 (2.25–2.51) | 2.21 (2.10–2.34) |
| CKD | 2.55 (2.43–2.69) | 2.44 (2.32–2.57) | 2.26 (2.15–2.38) | 2.13 (2.02–2.24) |
| All-cause death | 3.27 (3.11–3.43) | 2.71 (2.58–2.85) | 2.19 (2.09–2.31) | 1.93 (1.83–2.04) |
| Modified 3P-MACE | 2.29 (2.22–2.36) | 2.06 (2.00–2.12) | 1.82 (1.76–1.88) | 1.66 (1.61–1.71) |

**Table S12. Sensitivity analysis requiring two independent hypertension diagnoses**

| **Outcome** | **Events** | **Fully adjusted HR (95% CI)** |
| --- | --- | --- |
| Primary composite | 34,371 | 1.73 (1.69–1.78) |
| Myocardial infarction | 4,030 | 2.30 (2.13–2.47) |
| Stroke | 13,369 | 1.65 (1.59–1.72) |
| Heart failure | 8,400 | 2.19 (2.08–2.30) |
| CKD | 9,311 | 2.20 (2.09–2.31) |
| All-cause death | 9,677 | 1.34 (1.27–1.41) |
| Modified 3P-MACE | 24,147 | 1.57 (1.52–1.62) |

Exposure begins at the second independent hypertension diagnosis record, rather than the first diagnosis or qualifying prescription. There were 55,666 participants with a qualifying second diagnosis and 200,555 without. Main-analysis outcome definitions and adjustment covariates were retained.

**Table S13. Complete-case analysis**

| **Outcome** | **Events** | **Unadjusted HR (95% CI)** | **Fully adjusted HR (95% CI)** |
| --- | --- | --- | --- |
| Primary composite | 5,219 | 3.83 (3.61–4.07) | 2.49 (2.34–2.66) |
| Myocardial infarction | 572 | 3.45 (2.87–4.14) | 2.33 (1.93–2.82) |
| Stroke | 2,053 | 3.11 (2.82–3.43) | 1.91 (1.73–2.11) |
| Heart failure | 1,222 | 5.02 (4.39–5.75) | 3.17 (2.76–3.65) |
| CKD | 1,462 | 3.45 (3.06–3.88) | 2.47 (2.18–2.80) |
| All-cause death | 1,257 | 8.41 (7.34–9.64) | 4.54 (3.96–5.22) |
| Modified 3P-MACE | 3,544 | 4.19 (3.88–4.51) | 2.56 (2.37–2.77) |

The complete-baseline-data subset contains 38,970 participants. The event counts shown are the outcome-specific counts reported in the complete-case model output.

**Table S14. Primary composite under alternative missing-data approaches**

| **Approach** | **Analytic set** | **Fully adjusted HR (95% CI)** |
| --- | --- | --- |
| Multiple imputation | Full cohort | 2.49 (2.43–2.55) |
| Complete cases | Complete baseline data | 2.49 (2.34–2.66) |
| Missing/unknown categories | Full cohort | 2.52 (2.46–2.59) |

The missing-category model categorized continuous covariates into quintiles, with a separate missing/unknown category for variables with missing values. Complete-case and missing-category analyses assessed the robustness of estimates obtained after imputation.

**Table S15. Subgroup associations with the primary composite outcome**

| **Subgroup** | **Level** | **Adjusted HR (95% CI)** | **Interaction P** |
| --- | --- | --- | --- |
| Age | <65 years | 2.80 (2.71–2.89) | <0.001 |
| Age | ≥65 years | 2.10 (2.02–2.19) |  |
| Sex | Male | 2.47 (2.39–2.55) | <0.001 |
| Sex | Female | 2.58 (2.48–2.68) |  |
| Baseline SBP | <130 mmHg | 2.68 (2.58–2.78) | <0.001 |
| Baseline SBP | ≥130 mmHg | 2.37 (2.29–2.45) |  |
| Total cholesterol | <5.2 mmol/L | 2.52 (2.44–2.60) | <0.001 |
| Total cholesterol | ≥5.2 mmol/L | 2.49 (2.39–2.59) |  |
| HDL-C | Low | 2.51 (2.41–2.61) | <0.001 |
| HDL-C | Higher | 2.50 (2.43–2.58) |  |
| Glucose-lowering medication | No | 2.52 (2.39–2.65) | <0.001 |
| Glucose-lowering medication | Yes | 2.50 (2.43–2.57) |  |

HRs are from separately fitted subgroup models. Interaction P values are from the full cohort model with product terms. The full covariate set was retained except the stratifying variable. Low HDL-C is <1.0 mmol/L in men or <1.3 mmol/L in women.

**Figure S1. PH diagnostic plot for the primary composite**

Scaled Schoenfeld-residual diagnostic for the time-varying recorded-hypertension term in the primary-composite Cox model. The plotted coefficient varies with follow-up time. The hypertension-term and global Grambsch–Therneau tests both had P<0.001.
